# Identification of the full set of *Listeria monocytogenes *penicillin-binding proteins and characterization of PBPD2 (Lmo2812)

**DOI:** 10.1186/1471-2180-10-239

**Published:** 2010-09-15

**Authors:** Dorota Korsak, Zdzislaw Markiewicz, Gabriel O Gutkind, Juan A Ayala

**Affiliations:** 1 Institute of Microbiology, University of Warsaw, Miecznikowa 1, 02-096 Warsaw, Poland; 2 Facultad de Farmacia y Bioquimica, Universidad de Buenos Aires, Argentina; 3 Centro de Biologia Molecular "Severo Ochoa", Consejo Superior de Investigaciones Científicas, CSIC-UAM, C/Nicolás Cabrera 1, 28049, Madrid, Spain

## Abstract

**Background:**

Bacterial penicillin-binding proteins (PBPs) can be visualized by their ability to bind radiolabeled or fluorescent β-lactam derivatives both whole cells and membrane/cell enriched fractions. Analysis of the *Listeria monocytogenes *genome sequence predicted ten genes coding for putative PBPs, but not all of their products have been detected in studies using radiolabeled antibiotics, thus hindering their characterization. Here we report the positive identification of the full set of *L. monocytogenes *PBPs and the characteristics of the hitherto undescribed PBPD2 (Lmo2812).

**Results:**

Eight *L. monocytogenes *PBPs were identified by the binding of fluorescent β-lactam antibiotic derivatives Boc-FL, Boc-650 and Amp-Alexa430 to proteins in whole cells or membrane/cell wall extracts. The gene encoding a ninth PBP (Lmo2812) was cloned and expressed in *Escherichia coli *as a His-tagged protein. The affinity purified recombinant protein had DD-carboxypeptidase activity and preferentially degraded low-molecular-weight substrates. *L. monocytogenes *mutants lacking the functional Lmo2812 enzyme were constructed and, compared to the wild-type, the cells were longer and slightly curved with bent ends.

Protein Lmo1855, previously designated PBPD3, did not bind any of the antibiotic derivatives tested, similarly to the homologous enterococcal protein VanY.

**Conclusions:**

Nine out of the ten putative *L. monocytogenes *PBP genes were shown to encode proteins that bind derivatives of β-lactam antibiotics, thus enabling their positive identification. PBPD2 (Lmo2812) was not visualized in whole cell extracts, most probably due to its low abundance, but it was shown to bind Boc-FL after recombinant overexpression and purification. Mutants lacking Lmo2812 and another low molecular mass (LMM) PBP, PBP5 (PBPD1) - both with DD-carboxypeptidase activity - displayed only slight morphological alterations, demonstrating that they are dispensable for cell survival and probably participate in the latter stages of peptidoglycan synthesis. Since Lmo2812 preferentially degrades low-molecular- mass substrates, this may indicate a role in cell wall turnover.

## Background

Penicillin-binding proteins (PBPs) are responsible for the final synthesis steps of the universal peptidoglycan exoskeleton of bacteria. Since their initial identification by Brian Spratt [1] most attention has been paid to the activities of these proteins in model microorganisms such as *Escherichia coli, Bacillus subtilis *and *Streptococcus pneumoniae*.

The rise in resistance to β-lactam antibiotics and the diversity of the mechanisms involved, including modification of the target PBPs often seen in Gram-positive pathogens, has resulted in increased interest in this group of proteins.

*Listeria monocytogenes *causes relatively infrequent but often very serious food-borne infections termed listerioses, with mortality rates that can reach 25-30% [2-4]. Newborns and immunocompromised individuals are at special risk, and in these cases controlling the infection with antimicrobial agents can potentially be hindered due to the emergence of *L. monocytogenes *isolates with reduced susceptibility to ampicillin [5,6].

The penicillin-binding proteins (PBPs) of *L. monocytogenes *were first identified by Vicente *et al*. [7] using radiolabeled β-lactams, and it was subsequently suggested that PBP3 is the primary lethal target of these antibiotics [8,9]. However, as in many other bacteria, the exact mechanism of β-lactam-induced cell death remains unknown. There have been a limited number of reports dealing with the PBPs of *L. monocytogenes*. Earlier studies carried out in our laboratory - when only five PBPs were known - resulted in a re-estimation of the copy number of individual *L. monocytogenes *penicillin-binding proteins [10] and elucidation of the enzymatic properties of PBP4 (encoded by *lmo2229*) and PBP5 (*lmo2754*) [11-13]. A different approach to studying the penicillin-binding proteins of *L. monocytogenes *was made possible by the availability of the complete genome sequence of this bacterium [14]. The insertional mutagenesis of genes encoding seven potential PBPs -two of class A, three of the high molecular mass (HMM) class B and two of the low molecular mass (LMM) type - helped to clarify their role [15].

In the present study we have positively identified eight penicillin-binding proteins in whole cell extracts of *L. monocytogenes*, and another LMM PBP (Lmo2812) was characterized by the Bocillin-FL (Boc-FL)-binding ability of the purified recombinant protein.

## Results

### Detection and identification of *L. monocytogenes *PBPs

The "surfaceome" of the model *L. monocytogenes *strain EGDe has been annotated [14] and recently revised [16]. It includes proteins involved in the synthesis of peptidoglycan. Examination of sequence information from a database dedicated to the analysis of the genomes of *L. monocytogenes *(strain EGDe) and its non-pathogenic relative *Listeria innocua *(strain CLIP 11262) http://genolist.pasteur.fr/ListiList, as well as that from the Pfam database http://www.sanger.ac.uk/Software/Pfam and information from the NCBI Conserved Domain database http://www.ncbi.nlm.nih.gov/COG/ and the Interpro database http://www.ebi.ac.uk/interpro/, has identified 10 putative genes for PBPs, classified according to molecular class (Table 1).

**Table 1:** The full set of predicted PBPs in *L. monocytogenes*

**PBP**^***a***^	**PBP**^***b***^	**gene**^***c***^	**Class**^***d***^	Prototype	aa	MW (kDa)	IP	**Putative domain structure**^**e**^
PPBA1	PBP1	*lmo1892*	A-3	PBP1a (Spn)	827	90.87	9.15	SP-Φ-TG-TP

PBPB2	PBP2	*lmo2039*	B-4	PBP2x(Spn)	751	81.89	7.77	SP-Φ-D-TP

PBPB1	PBP3	*lmo1438*	B-5	PBP2b(Spn)	721	79.91	8.26	SP-Φ-D-TP

PBPA2	PBP4	*lmo2229*	A-4	PBP2a(Spn)	714	77.85	6.75	SP-Φ-TG-TP

PBPB3	-----	*lmo0441*	B-1	PBP2a(Sau)	678	74.60	6.57	SP-Φ-MecAN-D-TP

PBPD1	PBP5	*lmo2754*	C-T5	PBP3(Spn)	445	48.08	7.63	SP-CP-CA

PBPC1	-----	*lmo0540*	C-TH	AmpH(Eco)	397	44.53	9.70	SP-BLA

PBPC2	-----	*lmo1916*	C-TH	R61 (SR61)	335	37.84	7.04	BLA

PBPD3	-----	*lmo1855*	M15B	----	274	31.08	5.46	SP-CP(VanY)

PBPD2	-----	*lmo2812*	C-T5	PBP5 (Bsu)	272	29.48	4.59	SP(lipo)-CP

^*a*^Nomenclature of PBPs as defined in [16]; ^*b*^Nomenclature of PBPs as defined in [7,10]; ^*c*^gene names as identified in Listilist web server http://genolist.pasteur.fr/ListiList/; ^*d*^specific class of PBP as identified in [19]; ^e^domain structure of PBPs as described in [16]; SP, signal peptide; Φ, hydrophobic region; TG, transglycosylase domain; TP, transpeptidase domain; D, interaction domain; MecAN, homologous to PBP2a *S. aureus *resistance protein; CP, carboxypeptidase domain; CA, C-terminal anchor domain; BLA, β-lactamase domain; (VanY), homologous to VanY; SP(lipo), lipoprotein signal peptide.

**Figure 1:**
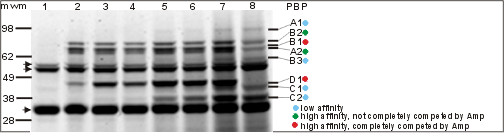
**Complete set of PBPs identified with Boc-FL in whole cells of *L. monocytogenes***. Samples of whole cells (100 μg of total protein) were labeled with Boc-FL at concentrations of 0 (1), 0.5 (2), 1 (3), 2.5 (4), 5 (5), 10 (6), 50 μM (7) and 50 μM plus 100 μg/ml ampicillin (8). Labeled bands were detected directly on the gel, quantified, and their molecular mass estimated. The affinity of each band for Boc-FL (ID50) was estimated from their fluorescence as a function of the concentration of Boc-FL. The name of the PBP corresponding to each band is indicated on the right, while the positions of molecular weight markers (bars) and unspecific bands (arrowheads) are shown on the left.

PBPs form a covalent complex with β-lactam antibiotics [1]. When fluorescent β-lactams are employed, these proteins can be visualized immediately following SDS-PAGE [17]. Total protein from whole cells or a cell wall extract of *L. monocytogenes *EGD were incubated with different concentrations of Boc-FL, Bocillin-650 (Boc-650) or Ampicillin-Alexa430 (Amp-430) for 30 min at 37°C. The highest affinity binding was obtained with Boc-FL and bands identified using this compound in the whole cell assay are shown in Figure 1. PBPs A1, B2, B1, A2, B3, D1, C1 and C2 were also identified with Boc-650 and Amp-430 (data not shown). Two types of non-specific band were also observed (lane 1, 0 μM Boc-FL) and they represent the natural intrinsic fluorescence of other proteins in the cell extract. However, the bands that are absent in lane 8 (ampicillin 100 μg/ml, 50 μM Boc-FL) compared with lane 7 (50 μM Boc-FL) represent specific PBPs. Those bands that completely disappeared (PBPB1, PBPD1), partially disappeared (PBPA1, PBPB2, PBPA2 and PBPB3) or remained present (PBPC1 and PBPC2) reflect total, partial and no binding of ampicillin, respectively. The results of an experiment examining saturation with 50 μM Boc-FL, the binding capacity of each PBP for Boc-FL and the affinity of the PBPs for ampicillin (Amp) are presented in Table 2. These assays involved incubation of whole cell with ampicillin followed by a similar incubation with Boc-FL. Therefore, only those PBPs with no or low affinity for ampicillin would be able to bind Boc-FL during the second incubation. The deacylation rate for the PBPs is actually extremely low, which permitted their detection in the gel for several hours after binding. Boc-FL binding to PBPs B1 and D1 was completely inhibited by Amp at 100 μg/ml, and these two PBPs exhibited high (Kd50 = 0.25 μM) and medium (Kd50 = 5.0 μM) affinity for Boc-FL, respectively. PBPs A1, B3, C1 and C2 showed low affinity (Kd50 >10 μM) for Boc-FL.

**Table 2:** Competition binding assay and affinity of different PBPs of *L. monocytogenes *for Boc-FL

PBP	**Boc-FL Kd50**^***a***^	**Ampicillin**^***c***^
PPBA1 (PBP1)	>10 μM	95

PBPB2 (PBP2)	0.25 μM	90

PBPB1 (PBP3)	0.25 μM	0

PBPA2 (PBP4)	0.25 μM	90

PBPB3	>20 μM	95

PBPD1 (PBP5)	5.0 μM	0

PBPC1	>20 μM	100

PBPC2	>20 μM	100

PBPD3	n.a.	n.a.

PBPD2	2.5 μM^*b*^	0^*b*^

^*a*^affinity of the respective bands for Boc-FL estimated from their fluorescence as a function of the concentration of Boc-FL (Kd50)

^*b*^obtained with purified recombinant Lmo2812

^*c*^percentage of Boc-FL binding capacity remaining after sample was preincubated with 100 μg/ml ampicillin

### Characterization of protein Lmo2812 (PBPD2)

Gene *lmo2812 *was amplified by PCR from the wild-type EGD strain and cloned in vector pET30a without its putative lipobox signal peptide. Expression of the His-tagged fusion protein in *E. coli *BL21(DE3) cells was induced with IPTG and it was purified from cell lysates on a nickel affinity column. The recombinant Lmo2812 protein was eluted from the column by washes with 250 and 500 mM imidazole. These two fractions were combined and further purified on a desalting column, yielding 4 mg/ml of pure protein.

**Figure 2:**
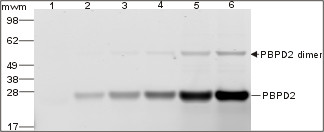
**Purified recombinant *L. monocytogenes *Lmo2812 (PBPD2) identified with Boc-FL**. Samples of purified recombinant Lmo2812 (10 μg) were labeled with Boc-FL at concentrations of 0 (1), 0.25 (2), 0.5 (3), 2.5 (4), 5 (5) and 10 μM (6). Labeled bands were detected directly on the gel, quantified and their molecular mass estimated. The affinity of the bands for Boc-FL (Kd50) was estimated from their fluorescence as a function of the concentration of Boc-FL. The names of the bands are indicated on the right, and the positions of the molecular weight markers are shown on the left.

The purified protein was incubated with different concentrations of Boc-FL (0.25, 0.5, 2.5, 5 and 10 μM). Saturation binding studies showed that Lmo2812 covalently bound Boc-FL, indicating that the recombinant protein retained its authentic activity. Lmo2812 was the major band on gels, with a slower migrating minor band thought to represent a dimeric form (Figure 2).

The β-lactam binding capacity of Lmo2812 was evaluated with three different fluorescent antibiotics: Boc-FL, Boc-650 and Amp-430. The purified protein was able to bind these compounds with apparent affinity constants (Kd50) of 2.5, 2.8 and 18.5 μM, respectively.

Since most LMM PBPs are DD-carboxypeptidases, the enzymatic activity of Lmo2812 was characterized in an *in vitro *assay using the synthetic tripeptide Nα,Nε-Diacetyl-Lys-D-Ala-D-Ala at concentrations of up to 12.5 mM as substrate with 40 μg of purified protein. The maximum activity was 0.75 pmoles/μg min, indicating low DD-carboxypeptidase activity under these assay conditions.

No β-lactamase activity could be detected in assays performed using the purified protein (data not shown).

The hydrolysis of whole peptidoglycan and purified natural muropeptides was also analyzed, but no such enzymatic activity was detected when the purified Lmo2812 (up to 100 μg of protein) was incubated for up to 18 h in the presence of 300 μg of whole peptidoglycan or up to 30 μg of the natural dimeric muropeptide D45 (NAcGlc-NAcMur-tetrapeptide-NAcGlc-NAcMur-pentapeptide). However, Lmo2812 was found to cleave the peptide bond between the subterminal and terminal D-alanine moieties (positions 4 and 5) of the pentapeptide side chain of the monomeric muropepeptide M5 (NAcGlc-NAcMur-pentapeptide) to convert the pentapeptide into a tetrapeptide M4 (NAcGlc-NAcMur-tetrapeptide). No such cleavage occurred in the absence of Lmo2812.

The pH-dependence of the activity of Lmo2812 against monomeric muropepeptide M5 was determined in the pH range of 4.5 to 7.0. The highest activity was detected in assays performed at pH 7.0 in a Tris-Mg buffer, where half of the substrate was converted to the tetrapeptide (Table 3).

**Table 3:** DD-carboxypeptidase activity of recombinant Lmo2812 using M5 muropeptide as the substrate

Reaction conditions	**M5 (%)**^**a**^	**M4 (%) **^**a**^
Lmo2812, M5, pH 4.5	97	3

Lmo2812, M5, Tris-Mg, pH 7.0	52	48

Lmo2812, M5, NaPi, pH 7.0	84	16

Control, M5, pH 7.0	99	1

^a^percentage of muropeptides M5 (NAcGlc-NAcMur-pentapeptide) and M4 (NAcGlc-NAcMur-tetrapeptide) determined by HPLC analysis

### Construction of single and double penicillin-binding protein mutants

Allelic exchange mutagenesis was used to create in-frame deletions in the *lmo2812 *and *lmo2754 *genes, which encode the penicillin-binding proteins Lmo2812 (PBPD2) and PBP5 (PBPD1), respectively. DNA fragments representing regions near the 5' and 3' ends of the genes were independently amplified, spliced, and inserted into the *E. coli - L. monocytogenes *shuttle vector pKSV7 to generate derivatives pKD2812 and pADPBP5, carrying the spliced regions of the *lmo2812 *and *lmo2754 *genes, respectively. *L. monocytogenes *cells transformed with these constructs were grown for several generations in TSBYE broth at 30°C in the presence of chloramphenicol to select for chromosomal integration of the plasmids. Excision of chromosomally-integrated plasmids was facilitated by repeated growth in the absence of antibiotic pressure, and a subsequent shift in the growth temperature was used to cure the cells of the excised plasmids. Colonies grown on TSBYE plates were screened for loss of chloramphenicol resistance and several sensitive clones were then examined by PCR to identify those in which an allelic exchange event had resulted in chromosomal replacement of the wild-type copy of the gene with the mutant allele. This first round of allelic exchange mutagenesis led to the isolation of the derivative *L. monocytogenes *KD2812, which had a 627-bp deletion in the *lmo2812 *gene.

The KD2812 single mutant was used in a second round of allele replacement mutagenesis, which began with the transformation of this strain with plasmid pADPBP5. Completion of the mutagenesis procedure led to the isolation of a double-mutant strain, *L. monocytogenes *AD07, which had a 627-bp deletion in the *lmo2812 *gene and a 1113-bp deletion in the *lmo2754 *(PBP5) gene.

### Characterization of KD2812 and AD07 mutant strains

To examine the effect of PBP deletion on cell growth rate, the doubling times of cultures of EGD, KD2812 and AD07 were determined. The doubling time of the wild-type strain grown at 37°C was 40 min, whereas those of the single and double mutants were 45 and 50 min, respectively. These data indicate that the single and double PBP deletion strains grew significantly slower (P < 0.05) than EGD. The doubling time of the double mutant was also significantly different from that of KD2812. Thus, although the bacteria were viable in the absence of Lmo2812 and PBP5, they grew more slowly than the wild-type.

**Figure 3:**
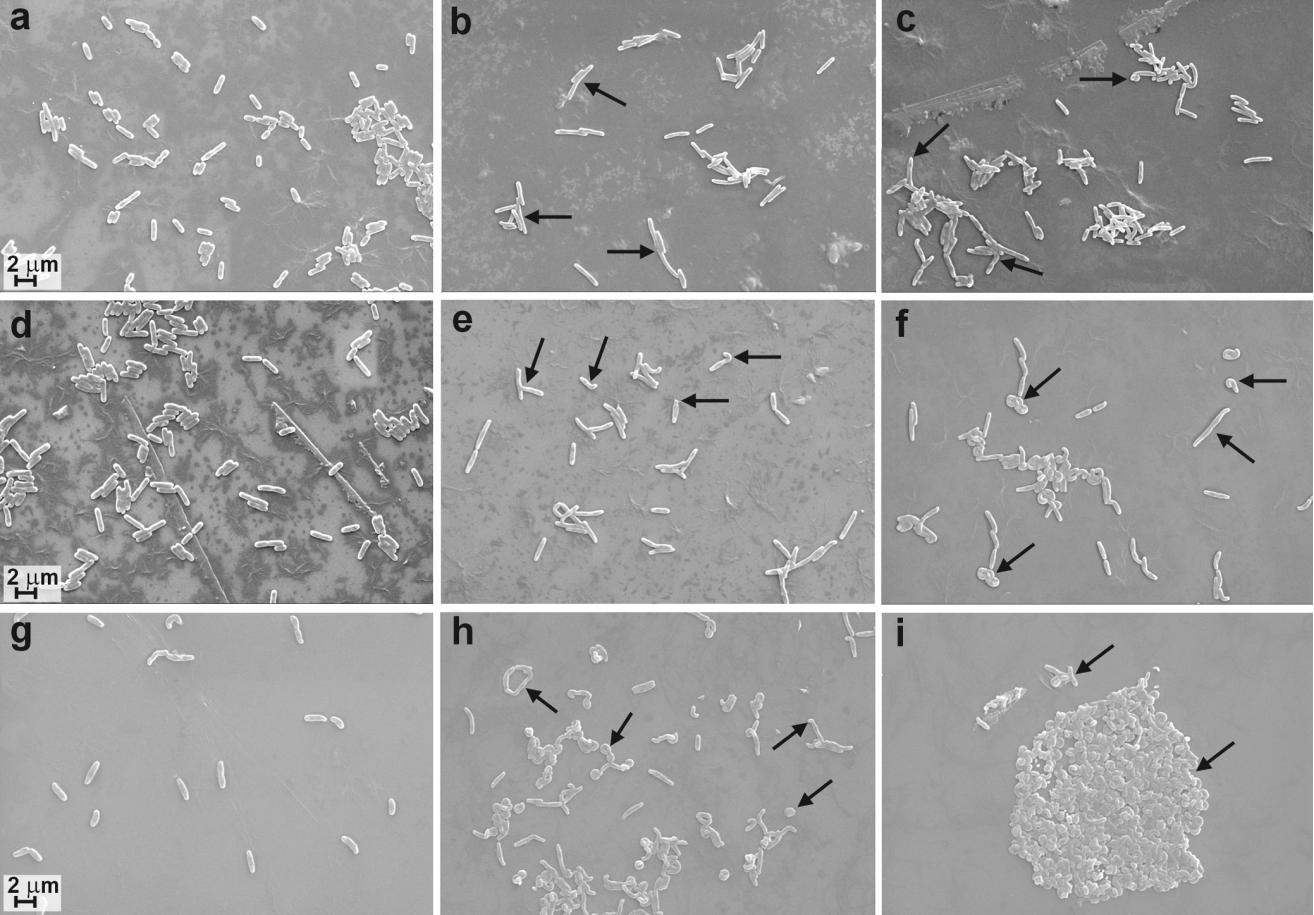
**Morphology of *L. monocytogenes *EGD and PBP mutants**. SEM images of cells of wild-type strain EGD (a, d, g), mutant KD2812 (b, e, h) and mutant AD07 (c, f, i). The growth temperatures of the cultures were 30°C (a to c), 37°C (d to f) and 42°C (g to i). Arrows indicate irregularly curved cells and increased cell length.

To determine the effect of these mutations on cell morphology, the strains EGD, KD2812 and DA07 were analyzed by scanning electron microscopy (SEM). As cells of the mutant strains displayed irregular morphology when grown at 42°C (Figure 3; h, i), the cell lengths were only determined when the strains were grown at 30 and 37°C. Cells of the *L. monocytogenes *strains lacking Lmo2812 were significantly longer than those of the wild-type (Student's *t *test, P < 0.05) (Table 4). At 30°C the average cell length compared to strain EGD was increased by 38.5% in strain KD2812 and by 44.8% in the double mutant strain. The respective values at 37°C were 37.5% and 43%. The populations of the single and double mutant strains also showed some variation in cell morphology. A proportion of the cells of strain KD2812 showed an altered phenotype at each of the tested temperatures. The variant cells were characteristically curved with a bend at either one or both ends and subterminal constrictions. The number of cells with altered morphology was increased as the growth temperature was raised (Figure 3; b, e, h). Cell bending was more pronounced in the population of AD07 mutant cells (Figure 3; c, f, i). More than 90% of cells of the double mutant exhibited irregular morphology at 42°C. To determine whether disruption of the PBP-encoding genes had an impact on the β-lactam resistance of *L. monocytogenes*, microdilution MIC tests were performed. The results demonstrated that the lack of functional *lmo2812 *and *lmo2754 *genes had little effect on the sensitivity of the mutant strains to the panel of β-lactam antibiotics tested (Table 5). Similar results were found for a mutant of *L. monocytogenes *lacking PBP5 (PBPD1) examined in a previous study [11].

**Table 4:** Cell length of *L. monocytogenes *EGD and mutant strains grown at different temperatures

Temperature	Strain	Average cell length (μm) ± SD	Minimum length/Maximum length (μm)	*n*
**30°C**	EGD	1.70 ± 0.38	0.99/3.80	245
	
	KD2812	2.35 ± 0.76	1.19/6.97	124
	
	AD07	2.46 ± 0.68	1.44/6.43	111

**37°C**	EGD	1.80 ± 0.44	1.05/3.64	150
	
	KD2812	2.48 ± 0.70	1.43/4.70	106
	
	AD07	2.581 ± 0.6	1.56/5.15	50

**Table 5:** MICs of some β-lactam antibiotics against *L. monocytogenes *EGD and mutant strains

Antimicrobial agent	MIC (μg/ml)		
	**EGD**	**KD2812**	**AD07**

penicillin	0.16	0.16	0.08
ampicillin	0.31	0.31	0.16
oxacillin	2.5	2.5	1.25
piperacillin	1.25	1.25	1.25
cefalotin	2	2	2
cefoxitin	32	32	32
cefotaxim	6	6	6
ceftazidime	256	256	256

**Figure 4:**
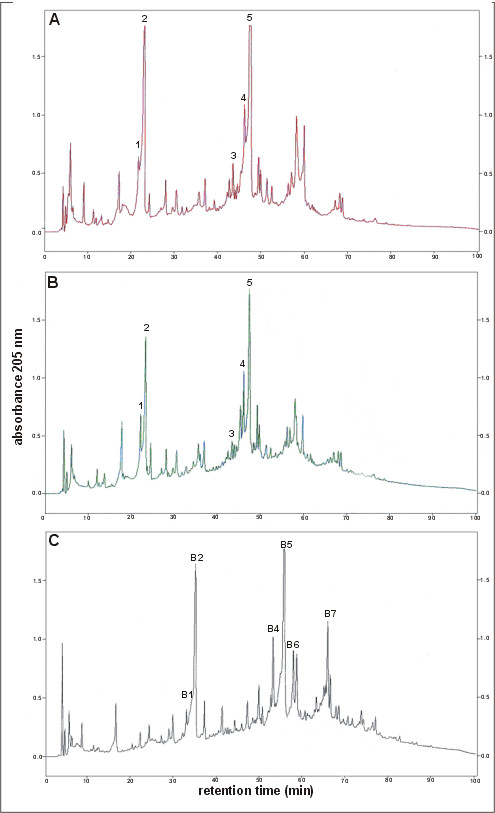
**HPLC elution patterns of muropeptides from wild-type and mutant *L. monocytogenes *peptidoglycan**. Muropeptides produced by the enzymatic hydrolysis of peptidoglycan purified from wild-type *L. monocytogenes *EGD (A), mutant KD2812 lacking functional Lmo2812 (B), and mutant AD07 lacking functional Lmo2812 and PBP5 (C), were reduced and separated by reversed phase HPLC and the *A*_205 _of the eluate was monitored: 1, 2 disaccharide-tripeptide monomers; 3,4,5 bis-disaccharide tri-tetra peptide dimers.

To compare the murein of *L. monocytogenes *mutants KD2812 and AD07 with that of the wild-type strain, muropeptides were released from isolated peptidoglycan by complete digestion with muramidase and the reduced muropeptides were analyzed by high performance liquid chromatography (HPLC) to that obtained for wild-type *L. monocytogenes*, but that of the double mutant was markedly different (Figure 4). Comparison of the peptidoglycan profiles of the wild-type strain and AD07 (Figure 4; A and 4C) indicated that both the composition and relative amount of a number of muropeptides were dramatically altered. All of the well characterized muropeptides identified in the murein of strain EGD, with tripeptide side chains in monomers or cross-linked muropeptides (e.g. muropeptides 1, 2, 3, 4, 5), were dramatically decreased or entirely absent in the double mutant. Furthermore, a number of novel muropeptides (B1 to B7) were detected in AD07 pepidoglycan. Peaks B1 and B2 may correspond to monomers with a disaccharide-pentapeptide structure, while B3-B7 may represent different forms of a dimer - a bis-disaccharide penta-tetra [18].

## Discussion

Previous analyses [7-10] of the *L. monocytogenes *cell membrane identified only five proteins able to bind I^125^-penicillinX, H^3^-benzylpenicillin, S^35^-penicillin or I^125^-ampicillin (PBP1, PBP2, PBP3, PBP4, PBP5), which correspond to PBPA1, B2, B1, A2 and D1, respectively. *In silico *analysis of the *L. monocytogenes *genome revealed the presence of ten open reading frames that potentially encode penicillin-binding proteins [16]. We believe that the present study is the first to have used fluorescently labeled antibiotics (Boc-FL, Boc-650 and Amp-430) to identify the PBPs of *L. monocytogenes*. With this method, we were able to identify eight PBPs, both in whole cell and membrane extracts. PBPB3, encoded by the gene *lmo0441*, was classified as a subclass B1 PBP [19]. All PBPs in this subclass, e.g. PBP2a of *Staphylococcus aureus *and PBP5 of *Enterococcus faecium*, are thought to exhibit low affinity for penicillin [20]. We found that PBPB3 also has low affinity for all the β-lactams tested. A recent study of seven *L. monocytogenes *genes encoding potential penicillin-binding proteins showed that interruption of the *lmo0441 *gene resulted in increased susceptibility of strain EGDe to β-lactams [15]. It was concluded that protein Lmo0441 (PBPB3) may play a central role in the β-lactam resistance of *L. monocytogenes *[15]. We identified two additional LMM PBPs, PBPC1 and PBPC2, which contain a β-lactamase class C domain. PBPC1 is predicted to be located at the surface of the bacterium, while PBPC2 lacks any recognized cell surface association domain [16]. However, we detected both proteins in intact cells, which indicates that some physical interaction of PBPC2 with the cell wall must exist.

The product of gene *lmo1855*, Lmo1855 (PBPD3), was not found to bind β-lactams with any of the various methods employed and consequently cannot be considered a PBP.

Lmo2812 (PBPD2), a low molecular mass PBP, has been identified as a class C type 5 protein related to the peptidase S11 family [19]. As Lmo2812 was not observed in Boc-FL-, Boc-650- and Amp-430-labeled extracts, it seemed possible that it does not bind β-lactam antibiotics. However, β-lactam binding experiments with purified recombinant protein demonstrated that Lmo2812 does bind the three different fluorescent antibiotics efficiently. The apparent affinity constants (Kd50) for Boc-FL, Boc-650 and Amp-430 were 2.5, 2.8 and 18.5 μM, respectively. The absence of an observable band corresponding to Lmo2812 following SDS-PAGE of the Boc-FL-labeled listerial extract cannot be due to lack of interaction with the β-lactam. This result suggests that *L. monocytogenes *grown in culture expresses this protein at a very low level. It has recently been shown that the two-component system CesRK controls the transcriptional induction of *lmo2812*. The expression of *lmo2812 *is positively regulated by CesR and inducible with ethanol and cefuroxime [21]. On the other hand, we were able to identify protein Lmo0441 in a whole cell extract using fluorescent-labelled antibiotics, and the expression of this PBP is also dependent on CesR [21].

Bacteria often have a major type-5 PBP which represents the most abundant LMM PBP they produce. The most highly expressed PBP in listerial membranes is PBP5. In a previous study we confirmed that PBP5 is a DD-carboxypeptidase that preferentially degrades low-molecular-weight substrates [11]. In the present study we found that PBP5 is also a protein with a high affinity for β-lactams.

*L. monocytogenes *produces one more type-5 PBP - Lmo2812 - but its role in cell wall biosynthesis and catalytic activity had not previously been examined. In this study, recombinant Lmo2812 was expressed in *E. coli *and purified in order to characterize its enzymatic activity and role in cell physiology. Lmo2812 lacking its signal sequence was expressed as a His-tagged fusion protein in the cytoplasm of *E. coli*, which allowed the purification of large amounts of functionally active protein.

Type-5 PBPs, with the exception of *S. aureus *PBP4, are strict DD-carboxypeptidases and are unable to catalyze transpeptidation reactions [19]. Using the synthetic tripeptide Nα,Nε-Diacetyl-Lys-D-Ala-D-Ala and the natural monomer NAcGlc-NAcMur-pentapeptide in an *in vitro *assay, we showed that Lmo2812 displays weak DD-carboxypeptidase activity, cleaving the peptide bond between the subterminal and terminal D-alanine moieties. However, the recombinant Lmo2812 was active against neither *E. coli *peptidoglycan nor the natural dimeric muropeptide D45 (disaccharide pentapeptide disaccharide tetrapeptide). This suggests that Lmo2812, like PBP5 [11], preferentially degrades low-molecular-weight substrates. Analysis of the muropeptide profiles of a *L. monocytogenes *mutant demonstrated that the lack of Lmo2812 activity does not affect the muropeptide structure of its peptidoglycan. However, the ratio of pentapeptides to tripeptides was found to be increased in cells lacking both Lmo2812 and PBP5. Similar changes have been observed in the peptidoglycan from a *L. monocytogenes *mutant lacking PBP5 [12], *B. subtilis *devoid of PBP5 [18] and *S. pneumoniae *with disrupted PBP3 activity [22]. These changes in the muropeptide profile indicate that *L. monocytogenes *PBP5, like PBP5 of *B. subtilis *and PBP3 of *S. pneumoniae*, is a DD-carboxypeptidase that plays a basic role in the maturation of the cell wall peptidoglycan.

Mutations in genes coding for low molecular mass PBPs are not lethal for the bacterial cell and in general these proteins seem to be redundant. Mutants can survive not only the lack of individual LMM PBPs, e.g. *Pseudomonas aeruginosa *[23], *S. pneumoniae *[24], *S. aureus *[25] and *Myxococcus xanthus *[26], but also the loss of all LMM PBPs, e.g. *E. coli *[27], *Neisseria gonorrhoeae *[28] and *B. subtilis *[29]. Similarly, we demonstrated that the inactivation of *L. monocytogenes *genes *lmo2812 *and *lmo2754 *is not lethal and these gene products are dispensable for the growth and survival of the cells.

The results of the present and previous studies indicate that the growth of *L. monocytogenes *is only slightly impaired when it lacks the activities of Lmo2812 or both Lmo2812 and PBP5 [11,12]. Reduced growth rates have also been reported for a *S. pneumoniae *mutant lacking functional PBP3 [24] and for a double *N. gonorrhoeae *mutant lacking both PBP3 and PBP4 [28]. On the other hand, no changes in growth rate were observed for *E. coli *or *B. subtilis *mutants lacking most or all of their DD-carboxypeptidase activity [27,29].

However, the loss of Lmo2812 did result in significant changes in morphology. The mutant cells were significantly longer, slightly curved and had bent ends. These changes were even more pronounced in the double mutant AD07 lacking both Lmo2812 and PBP5. This finding is interesting because we did not notice any alterations in cell shape in a *L. monocytogenes *mutant lacking PBP5 alone, although the cell wall of the mutant was much thicker than that of the parental strain [11,12], even though Guinane *et al*. [15] did describe such changes. The differences between our observations may be due to variation in the strain (EGD versus EGDe) or growth conditions employed [15].

The reason for the prominent morphological changes in strain KD2812 is difficult to pinpoint since there do not seem to be any remarkable changes in the muropeptide structure of the peptidoglycan of this mutant. However, the observed changes in cell morphology implicate the protein in the late stages of peptidoglycan synthesis, presumably in the determination of the availability of pentapeptide substrates. Our finding that Lmo2812 preferentially degrades low-molecular-weight substrates may point to the a role for this protein in cell wall turnover. Further studies are required to clarify the function of Lmo2812, although, as in the case of extensive studies on the D-alanine carboxypeptidases of *E. coli *[30] and other bacteria, they may not yield conclusive results.

## Conclusions

The results of this study conclusively show that nine of the ten previously identified putative PBP genes of *L. monocytogenes *code for proteins that bind β-lactam antibiotics and their labeled or fluorescent derivatives. Eight of these proteins were identified in whole cell extracts, whereas the ninth protein, Lmo2812, was only shown to bind β-lactams following expression in *E. coli *and subsequent purification by affinity chromatography. The inability to detect Lmo2812 activity in the *L. monocytogenes *cell may be explained by the low abundance of this protein, whose expression is regulated by the two-component system CesRK [21]. We have also demonstrated that the LMM PBP Lmo2812 is a DD-carboxypeptidase and has no discernible β-lactamase activity. Mutants lacking the protein grow normally, although their cells are often longer and slightly curved. Similar morphological changes were observed in the case of a double mutant lacking two LMM carboxypeptidases: Lmo2812 and Lmo2754. Our results indicate that Lmo2812 most probably participates in the late stages of peptidoglycan synthesis, in the determination of the availability of pentapeptide substrates. Moreover, the fact that Lmo2812 preferentially degrades low-molecular-weight substrates may point to a role in cell wall turnover. The product of the tenth putative PBP gene, Lmo1855, was not found to bind β-lactams with any of the various methods employed and consequently cannot be considered a PBP. In this respect it resembles the homologous protein VanY from VanA- and VanB-type enterococcal strains. This study extends the number of identified penicillin-binding proteins from the original five [7,10] to the final number of nine which represents the full set of these proteins in *L. monocytogenes*.

## Methods

### Strains, plasmids and growth conditions

*E. coli *BL21(DE3) and DH5α were grown aerobically at 37°C on Luria-Bertani (LB) medium. *L. monocytogenes *strains were grown on Tryptic Soy Broth Yeast Extract (TSBYE) and Brain Heart Infusion (BHI) media at 37°C unless otherwise stated. Plates of solid LB or TSBYE media were prepared following the addition of agar to 1% (w/v). Ampicillin (100 μg/ml) or kanamycin (30 μg/ml) and chloramphenicol (10 μg/ml) were added to broth or agar media as required. When necessary, 0.1 mM IPTG (isopropyl β-D-1-thiogalactopyranoside) and X-Gal (5-bromo-4-chloro-3-indolyl-b-D-galactopyranoside) (20 μg/ml) were spread on agar plates 30 min prior to plating. The bacterial strains, plasmids and oligonucleotide primers used in this study are shown in Tables 6 and 7.

**Table 6:** Strains and plasmids used in this study

Strain or plasmid	Relevant genotype and features	Reference or source
**strains**		

EGD	*L. monocytogenes *wild-type	

KD2812	Δ*lmo2812 *derivative of EGD	This work

AD07	Δ*lmo2754 *derivative of KD2812	This work

*E. coli *DH5α	F^- ^Φ80 Δ *lac*ZM15(l*ac*ZYA-*org*F) U169 *deo*R *rec*A1 *end*A1 *hsd *R17 *pho*A *sup*E44kλ^-^*thi*-1 *gyr*A96 *rel*A1	

*E. coli *BL21(DE3)	F^- ^*omp*T *gal dcm hsd*S_B_(r_B_^- ^m_B_^-^) λ(DE3)	Novagen

**plasmids**		

pET30a		Novagen

pAD3	pET30a derivative containing *lmo2812 *gene	This work

pKSV7	temperature-sensitive integration vector; MCS^*a*^; *lac*Z; β-lac; *cat*, pE194 Ts rep	[31]

pKD2812	pKSV7 carrying the Δ*lmo2812 *allele	This work

pADPBP5	pKSV7 carrying the Δ*lmo2754 *allele	This work

^*a*^MCS - multiple cloning site

**Table 7:** Oligonucleotide primers used in this study

primer	Sequence 5'→3'
pET6up3^*a*^	AGCAAATCATATGGCGGTTTATTCAGTCG
pET6down^*a*^	ATGCTCGAGATCTTCTTTAAACCCAACCTC
La2812	ATCCGCTATCTGAATCGCCT
Pb2812^*b*^	TTCAGCTGTTCCAATTATTGCTCCGTAGAACAGGCTG
Lc2812	TTGGAACAGCTGAACGTGGA
Pd2812	CTAGAGTCAATCCGCAGCCA
La2754	CCGTTATTGACATCTGCTAC
Pb2754^*b*^	CCGCAGAAGCACCAATAACTGCCAGCGACGTTGAA
Lc2754	TTGGTGCTTCTGCGGCTTGT
Pd2754	TAGCAGATGGCATCATCCGG

^*a *^Nucleotide substitutions to create restriction sites are underlined

^*b *^Overhangs complementary to SOE primers are underlined

### Construction of *L. monocytogenes *mutant strains

#### (i) Construction of the Δ*lmo2812 *single mutant

The splicing by overlap extension (SOE) PCR approach was used to create an internal deletion construct for the *lmo2812 *gene. Primers La2812 and Pb2812 (Table 7) were used to amplify a 545-bp fragment comprising the 5' end of *lmo2812*, and primers Lc2812 and Pd2812 were used to amplify a 522-bp fragment comprising the 3' end of this gene from genomic DNA *L. monocytogenes EGD*. The two fragments were purified and used as the templates in a third PCR with primers La2812 and Pd2812, which generated a Δ*lmo2812 *allele with a 627-bp deletion extending from nucleotides +73 to +700. Deletions in the gene *lmo2754 *were constructed by a similar approach using SOE primers shown in Table 7. The Δ*lmo2754 *allele has a 1113-bp deletion (extending from nucleotides +86 to +1219). The Δ*lmo2812 *and Δ*lmo2754 *alleles were ligated as blunt-ended fragments to SmaI-digested *E. coli*-*L. monocytogenes *shuttle vector pKSV7 [31] and used to transform *E. coli *DH5α to generate plasmids pKD2812 and pADPBP5, respectively. pKD2812 was introduced into *L. monocytogenes *EGD by electroporation [32] and transformants were selected on TSBYE plates containing 10 μg/ml chloramphenicol. The transformants were grown briefly at 30°C and then plated on TSBYE plus chloramphenicol and grown at 42°C to select for integration of the plasmid by homologous recombination. Colonies with a chromosomal integration were then serially propagated in TSBYE without chloramphenicol at 30°C. Single clones were picked and replica plated on TSBYE and TSBYE plus chloramphenicol to identify those having undergone excision and loss of the plasmid. The presence of the desired allelic exchange in chloramphenicol-sensitive colonies was then confirmed by PCR using primers La2812 and Pd2812. The resulting mutant strain with a deletion in the *lmo2812 *gene was designated KD2812.

#### (ii) Construction of a Δ*lmo2812 *Δ*lmo2754 *double mutant

A double mutant strain was constructed by introducing the pKSV7 derivative pADPBP5 into *L. monocytogenes *KD2812 by electroporation. This was followed by the integration excision, curing and screening steps described above. The desired allelic exchange event was confirmed by PCR using the primers La2754 and Pd2754, and a PBP assay. The resulting mutant strain with deletions in the *lmo2812 *and *lmo2754 *genes was designated AD07.

### Inducible expression of recombinant Lmo2812 protein

Recombinant expression experiments were performed with *E. coli *BL21(DE3) harboring a derivative of the vector pET30a (Novagen). The *lmo2812 *gene without its signal sequence was amplified from *L. monocytogenes *EGD genomic DNA using primers designed from its sequence in GenBank (accession number AL591984). The upstream primer pET6up3 (Table 7) annealed to *lmo2812 *codons 33-38 and contained an in-frame NdeI restriction site at the 5'-end and a translation initiation codon in frame with the triplet coding for the first residue of the mature Lmo2812, whereas the downstream primer pET6down annealed to the last seven codons of the coding sequence and contained a XhoI site at the 5'-end. The PCR thermocycle was performed using a gene cycler (Amersham Biotech) and consisted of an initial denaturation for 5 min (94°C) followed by 30 cycles of amplification (30 s at 94°C, 30 s at 55°C and 45 s at 72°C) and a final extension for 10 min at 72°C. The amplified fragment was digested with NdeI and XhoI and cloned into vector pET30a that had been digested with the same endonucleases, which fused *lmo2812 *with a sequence encoding a hexahistidine peptide. The cloned insert was sequenced and found to be identical to the *lmo2812 *sequence in the completed EGDe genome (accession number AL591984). The expression plasmid pAD3 (pET30a-*lmo2812*) was used to transform *E. coli *BL21(DE3).

### Overexpression and purification of a soluble recombinant form of Lmo2812

For the expression of recombinant Lmo2812 protein, an overnight culture of strain BL21(DE3) harboring the plasmid pAD3 was diluted 1:100 into 1 litre of LB medium and this was incubated with shaking at 37°C. When the OD_600 _reached 0.6, IPTG (isopropyl β-D-1-thiogalactopyranoside; Sigma, 1 mM) was added and the culture was shaken at 37°C for 24 hours. The culture was cooled on ice and the cells were then harvested by centrifugation (7000 × *g*, 15 min, 4°C). All subsequent steps in the purification of the protein were performed at 4°C. The cell pellet was resuspended in 50 mM sodium phosphate buffer (NaPi), pH 8.0 containing 0.3 M NaCl and 0.1% Tween-20. After adding DNase (10 μg/ml) and phenylmethanesulfonyl fluoride (1 mM), the cells were broken by sonication (VCX-600 ultrasonicator Sonics and Materials, USA). Cell debris was removed by centrifugation (7000 × *g*, 15 min, 4°C). and the cell lysate supernatant containing the fusion protein was applied to a 5 ml nickel column according to the manufacturer's instructions (Qiagen). The column was washed with wash buffer (50 mM NaPi buffer pH 8.0, 0.3 M NaCl, 20 mM imidazole, 10% glycerol). The bound proteins were then eluted with a 50 mM 1 M gradient of imidazole in elution buffer (50 mM NaPi buffer pH 8.0, 0.3 M NaCl) at a flow rate of 40 ml/h. Protein purity was determined by SDS-PAGE. Fractions 9-10 (2.5 ml each) containing recombinant Lmo2812 were combined and further purified on an Econo-Pac 10 DG (Bio-Rad) desalting column against column running buffer (50 mM NaPi buffer pH 7.0, 50 mM NaCl), following the manufacturer's instructions.

### Fluorescent antibiotic binding assay

Total whole cell proteins or purified recombinant protein resuspended in 50 mM NaPi buffer, pH 7.0 were labeled by incubation at 37°C for 30 min with different concentrations of Boc-FL (Molecular Probes), Boc-650 (Molecular Probes) or Amp-430 (prepared in the laboratory by coupling ampicillin to Alexa-430), and then separated on a 10% acrylamide, 3.3% cross-linkage SDS-PAGE gel. To avoid degradation of the fluorescent β-lactam antibiotics by β-lactamases, samples were incubated at 37°C with clavulanic acid at a final concentration of 10 μg/ml or EDTA at a final concentration of 10 mM for 30 min before labeling, where appropriate. Competition experiments were carried out by preincubation of the samples with ampicillin (100 μg/ml) for 30 min at 37°C, before adding the fluorescent antibiotic. The samples were then further incubated for 30 min at 37°C. PBPs were visualized directly on the polyacryloamide gel by fluorescence using a Typhoon 9410 imager (Amersham Biosciences) with excitation wavelengths of 588, 633 or 457 nm and emission filters 520BP40, 670BP30 or 555BP20 for Boc-FL, Boc-650 and Amp-430, respectively.

Affinity constants for the binding of the labeled β-lactase to recombinant Lmo2812 were calculated from the results of binding assays using increasing concentrations of protein and/or antibiotic, and from the binding curves, apparent Kd values were determined as the concentration of antibiotic required for 50% of maximum binding.

### β-lactamase activity assay

β-lactamase activity was determined using the nitrocefin test (Oxoid) and quantified with 0.10 mM nitrocefin in 50 mM NaPi (pH 7.0, 22°C) by a spectrophotometric method. Nitrocefin (50 μg/ml) and 10 μl of extract were incubated for 1 h in a final volume of 500 μl at room temperature in 50 mM NaPi pH 7.0 (22°C). The absorbance was measured at 486 nm.

### DD-carboxypeptidase activity assay

A modification of the method of Frere *et al*. [33] was used for DD-carboxypeptidase activity measurement. A reaction mixture comprised of 15 μl of Nα,Nε-Diacetyl-Lys-D-Ala-D-Ala (25 mM), 3 μl of buffer (300 mM Tris-HCl pH 7.5) and 12 μl of purified recombinant Lmo2812 was prepared, incubated at 37°C and samples were taken every 10 min for 1 h. To these samples, 5 μl of 10 mg/ml (in methanol) o-Dianisidine (SIGMA) and 70 μl of enzyme/coenzyme mix (flavinadenine dinucleotide (FAD), Peroxidase and D-Amino acid Oxidase) were added. These mixtures were incubated at 37°C for 5 min, then 400 μl of methanol-water (v/v) was added and incubation continued at 37°C for another 2 min. The absorbance of each reaction was immediately read at 460 nm. A number of controls were performed: reactions containing only recombinant Lmo2812 fractions, reactions lacking recombinant Lmo2812 to establish the level of natural degradation of the tripeptide for at each sampling point, and standard samples containing known amounts of D-alanine.

### Enzymatic activity assay with natural muropeptides

Whole total peptidoglycan and purified muropeptides were isolated from *E. coli *cells as described previously [34]. A 10 μg sample of recombinant Lmo2812 was mixed with 5 μg of M5 (NAcGlc-NAcMur-pentapeptide) or D45 (NAcGlc-NAcMur-tetrapeptide-NAcGlc-NAcMur-pentapeptide) in a volume of 30 μl using three different buffer conditions: pH 4.5 (50 mM NaPi, 1% methanol, pH 4.5), pH 7.0 (30 mM Tris-HCl, 3 mM MgCl_2_, pH 7.0), or NaPi (50 mM sodium phosphate buffer, pH 7.0). These mixtures were incubated at 37°C for 120 min. Control samples of M5 or D45 without Lmo2812 were similarly incubated in 30 mM Tris-HCl buffer, 3 mM MgCl_2_, pH 7.0. The samples were analyzed by HPLC using a C18 reversed phase column and a methanol gradient to separate the peaks.

### Preparation of *L. monocytogenes *cell wall peptidoglycan

An overnight culture of the required strain (200 ml) was cooled on ice and the cells harvested by centrifugation (7000 × *g*, 10 min, 4°C). The cell pellet was resuspended in 1/40th of the original culture volume of 50 mM Tris-HCl buffer, pH 7.5. Glass beads (diameter 150-215 μm; Sigma) were added to the cell suspension (1 g per ml) prior to sonication using a VCX-600 ultrasonicator (Sonics and Materials, USA) for ten 1 min bursts at an amplitude of 20%. Unbroken cells were pelleted by centrifugation (7000 × *g*, 10 min, 4°C) and the supernatant was collected and mixed with an equal volume of hot 8% (v/v) sodium dodecyl sulfate (SDS). This mixture was boiled for 30 min and the resulting insoluble cell wall preparation was collected by centrifugation (150,000 × *g*, 30 min, 22°C) and washed with hot distilled water (60°C) at least five times to remove SDS. The SDS-free material was treated with α-amylase (100 μg/ml) for 2 h at 37°C, after which pronase E (200 μg/ml) was added and the incubation continued for 90 min at 60°C. Trichloroacetic acid was then added to a final concentration of 5% and the cell wall suspension was incubated for 24 h with stirring at 4°C to remove teichoic acid. The remaining insoluble material was collected by centrifugation (150,000 × *g*, 30 min, 4°C) and washed with cold distilled water until the pH became neutral. *N*-acetylation of murein was performed using acetic anhydride in the presence of NaHCO_3 _according to the method of Hayashi *et al*. [35]. The prepared peptidoglycan was stored at -20°C.

### Enzymatic hydrolysis of peptidoglycan and HPLC separation of soluble muropeptides

Prepared *L. monocytogenes *peptidoglycan samples (300 μg) were digested with the muramidase Cellosyl (Hoechst AG) as previously described [12]. Soluble muropeptides were reduced by treatment with sodium borohydride. The reaction was stopped after 30 min by lowering the pH to 3.5 with phosphoric acid. The reduced muropeptides were analyzed by HPLC on a Hypersil octadecylsilane (ODS) reversed-phase column (250 mm × 4 mm, particle size 3 mm diameter; Teknochroma) according to the method of Glauner [34]. The elution buffers used were 50 mM sodium phosphate containing 0.8 g/l sodium azide, pH 4.35 (buffer A) and 15% methanol in 75 mM sodium phosphate, pH 4.95 (buffer B). Elution conditions were 7 min isocratic elution in buffer A, 115 min of linear gradient to 100% buffer B and 28 min of isocratic elution in buffer B. The flow rate was 0.5 ml/min and the column temperature was 35°C. Eluted compounds were detected by monitoring the A_205_.

### Scanning electron microscopy

Small cultures (10 ml) of *L. monocytogenes *EGD, KD2812 and AD07 were grown at 30, 37 or 42°C in BHI medium to an OD_600 _of 0.6 and then harvested by centrifugation at (7000 × *g*, 10 min, at room temeprature). The cells were fixed for 30 min in 4% paraformaldehyde, washed three times in phosphate-buffered saline, pH 7.4, then dehydrated using a graded ethanol series (25, 50, 75, 96% ethanol; 15 min for each step). One drop of cell suspension was spread on a microcover, coated with gold, and examined using a LEO 1430VP scanning electron microscope (SEM).

### Antibiotic susceptibility tests

Microdilution tests were performed using cation-adjusted Mueller-Hinton broth (CAMHB) supplemented with 5% lysed horse blood containing two-fold dilutions of the antimicrobial agents. These mixtures were dispensed in 100 μl aliquots into plastic 96-well plates. To prepare inocula, a single colony of each strain from a TSBYE plate was transferred into 10 ml of the same medium and incubated for 24 h at 37°C. These cultures were serially diluted in CAMHB to a concentration of 10^5 ^cfu/ml and 100 μl aliquots were added to the microdilution plates. The plates were incubated for 18-20 h at 37°C before the reading of the MIC endpoints. The MIC was the lowest antibiotic concentration at which visible growth was inhibited.

## Authors' contributions

DK carried out the molecular cloning, recombinant protein expression and protein purification as well as the physiological characterization of the obtained mutants, and helped to draft the manuscript. ZM conceived part of the study, participated in its design and coordinated the preparation of the manuscript. GOG conceived part of the study and collaborated in preparation of the manuscript. JAA carried out the purification of natural muropeptides, binding assays in whole cells, purified recombinant proteins and performed enzymatic assays with natural and synthetic substrates. He also participated in the design of the experiments and the preparation of the manuscript. All authors read and approved the final version of manuscript.
